# Biocompatibility study of a silk fibroin-chitosan scaffold with adipose tissue-derived stem cells *in vitro*

**DOI:** 10.3892/etm.2013.1185

**Published:** 2013-06-26

**Authors:** WENCHEN JI, YUELIN ZHANG, SHOUYE HU, YONGTAO ZHANG

**Affiliations:** 1 Medical School of Xi’an Jiaotong University, Xi’an 710061;; 2 Department of Neurosurgery, The Third Affiliated Hospital, Medical School of Xi’an Jiaotong University, Xi’an 710068, P.R. China

**Keywords:** adipose stem cells, silk fibroin-chitosan scaffold, biocompatibility

## Abstract

The use of tissue engineering technology in the repair of spinal cord injury (SCI) is a topic of current interest. The success of the repair of the SCI is directly affected by the selection of suitable seed cells and scaffold materials with an acceptable biocompatibility. In this study, adipose tissue-derived stem cells (ADSCs) were incorporated into a silk fibroin-chitosan scaffold (SFCS), which was constructed using a freeze-drying method, in order to assess the biocompatibility of the ADSCs and the SFCS and to provide a foundation for the use of tissue engineering technology in the repair of SCI. Following the seeding of the cells onto the scaffold, the adhesion characteristics of the ADSCs and the SFCS were assessed. A significant difference was observed between the experimental group (a composite of the ADSCs with the SFCS) and the control group (ADSCs without the scaffold) following a culture period of 6 h (P<0.05). The differences in the results at the following time-points were statistically insignificant (P>0.05). The use of an MTT assay to assess the proliferation of the cells on the scaffold revealed that there were significant differences between the experimental and control groups following culture periods of 2 and 4 days (P<0.05). However, the results at the subsequent time-points were not statistically significantly different (P>0.05). Scanning electron microscopy (SEM), using hematoxylin and eosin (H&E) staining, was used to observe the cellular morphology following seeding, and this revealed that the cells displayed the desired morphology. The results indicate that ADSCs have a good biocompatibility with a SFCS and thus provide a foundation for further studies using tissue engineering methods for the repair of SCI.

## Introduction

Spinal cord injury (SCI) is a global problem that may lead to permanent disability. As such, the reversal of SCI has become the focus of numerous studies. In recent years, tissue engineering, as a new technology, has been utilized in a number of areas in bioscience. There are three key elements of tissue engineering: seed cells, scaffold materials and growth factors (1). The use of this technology to repair SCI is, at present, a key subject of scientific study (2). The predominant factor that determines the success of the repair process is the interaction between the seed cells and the scaffold (3,4); therefore, the selection of the appropriate cells and scaffold with the desired biocompatibility is crucial.

Since adipose tissue-derived stem cells (ADSCs) are readily accessible and demonstrate rapid proliferation, they are commonly used as seed cells (5,6). In tissue engineering technology, the scaffold acts as an artificial extracellular matrix (ECM); it provides a template that supports cell attachment, guides cell proliferation and differentiation and acts as a carrier for the transportation of cells to the site of the defect (7). A number of natural and synthetic polymers are currently being used as scaffold materials. These include silk-fibroin (SF), which possesses desirable mechanical properties, biocompatibility and the ability to support several cell types, but when dry is brittle and challenging to handle (8,9). Another polymer that has been investigated for use as a scaffold in tissue engineering is chitosan (CS); its biocompatibility, biodegradability and toxicity have been extensively investigated. However, pure CS scaffolds have been demonstrated to degrade rapidly and display a high-swelling property in aqueous solution (10). In order to avoid the problems presented by these individual polymers, the blending of the materials has been suggested (11,12). Silk fibroin-chitosan scaffolds (SFCSs) have been demonstrated to possess desirable mechanical properties and may therefore be used in the repair of SCI (13,14). However, the biocompatibility of ADSCs with SFCSs remains largely unexplored.

The aim of the current study was to extract, identify and differentiate ADSCs from Sprague-Dawley (SD) rats and to assess their biocompatibility with SFCSs. Furthermore, the study aimed to provide a foundation for further experiments investigating the use of tissue engineering technology in the repair of SCI.

## Materials and methods

### Experimental animals

Three-month-old SD rats, weighing 300±50 g, were provided by the Experimental Animal Center of the Medical School of Xi’an Jiaotong University (Xi’an, China). All the experiments were approved and supervised by the Ethics Committee of Xi’an Jiaotong University.

### Isolation and culture of ADSCs

Pentobarbital sodium (40 mg/kg; Sigma, St. Louis, MO, USA) was used to anesthetize the rats. The adipose tissue was then harvested, sheared into pieces and incubated for 1 h with 0.1% type I collagenase (Sigma) in a centrifuge tube containing Dulbecco’s modified Eagle’s medium (DMEM)/F12 (Sigma) with 20% fetal bovine serum (FBS; Hyclone, Logan, UT, USA) at 37°C. Following this, the mixture was centrifuged at 3,000 × g for 12 min, the supernatant was discharged and phosphate-buffered saline (PBS; Sigma) was added. This was subsequently centrifuged at 1,200 × g for 8 min, prior to the sediment being suspended with DMEM/F12. The cell density was then adjusted to 1×10^5^ / ml and the cells were seeded in a 25 cm^2^ cell culture flask, which was placed into a cell culture box at a temperature of 37°C, 5% CO_2_ concentration and 95% humidity. When the original cell generation reached 90% confluence, the entire medium was aspirated and the cells were washed with prewarmed PBS. This was achieved by pipetting the solution over the cell layer several times, in order to clean the cells thoroughly of any tissue fragments and blood cells. Following this, 0.25% trypsin/0.02% EDTA solution (Sigma) was added to the cells. The process was observed using an inverted phase contrast microscope, and when the cells detached from the culture flask, DMEM/F12 was added to terminate the digestion. Following this, centrifugation was performed at 1,700 × g for 10 min. The supernatant was then aspirated and the cells were suspended with DMEM/F12 (containing 20% FBS), to enable the passage of cells according to a 1:3 ratio.

### Induction of the cells into adipogenic and osteogenic directions

The third generation of cells were seeded at a density of 1×10^4^ /well in six-well plates, prior to being divided into control and experimental groups. When the cells reached 70% confluence, the osteogenic experimental group was placed in an osteogenic induction medium consisting of 10% FBS/L-DMEM, 0.1 *μ*mmol/l dexamethasone, 10 mmol/l sodium β-glycerol phosphate, 50 mg/l vitamin C and 0.01 *μ*mol/l vitamin D_3_ (Sigma). The control group was maintained in DMEM/F12 (containing10% FBS), and, after 2 and 4 weeks, alkaline phosphatase and Alizarin Red staining were performed, respectively. The adipogenic experimental group was placed in an adipogenic induction medium consisting of 10% FBS/L-DMEM, 0.1 *μ*mmol/l dexamethasone, 10 *μ*g/ml insulin, 0.5 mmol/l 3-isobutyl-1-methylxanthine (IBMX) and 0.01 *μ*mmol/l indomethacin (Sigma), while the corresponding control group was maintained in DMEM/F12 (containing 10% FBS). Oil Red O staining (Xi’an chemical reagent factory, Xi’an, China) was conducted after 2 weeks.

### Incorporation of the ADSCs into the SFCS and the assessment of the cell adhesion rate

The SFCS (50% SF and 50% CS) was made using a freeze drying method and was subsequently sterilized with ethylene oxide, prior to the application of a polylysine coating (Sigma). The SFCS was placed into a 96-well plate and was soaked with cell culture liquid. Third generation ADSCs were obtained, and the cell density was adjusted to 1×10^6^ / ml. The cells were then divided into control and experimental groups. In the experimental group, 200 *μ*l cell suspension fluid was added to each scaffold, prior to the scaffold being rested for 4 h. Following this, a further 2 ml cell suspension fluid (cell density, 1×10^6^ /ml) was added into each well. The control group consisted solely of cells, without the scaffold. The experimental and control groups were placed into the cell culture box (37°C, 5% CO_2_ concentration and 95% humidity), and the cell culture solution was replaced every 3 days. Following 6, 12, 18 and 24 h, prewarmed PBS was used to wash the SFCS several times, in order to remove the cells that had not adhered, and the cells had had adhered to the scaffold were digested with 0.25% trypsin/0.02% EDTA solution. The cell adhesion rate was calculated according to the following formula: Cell adhesion rate (%) = number of adhered cells / total number of cultured cells × 100. At each time point, seven wells were used to determine the mean and standard deviation values for the two groups (experimental and control).

### Assessment of cell proliferation rate

The experimental group consisted of a composite of the ADSCs with the SFCS, while the control group consisted solely of cells. The cell culture medium was exchanged every 2 days. Cell culture plates were obtained following 2, 4, 6, 8 and 10-day culture periods. The entire medium was aspirated, prior to the addition of 1 ml DMEM/F12 cell culture liquid and 200 *μ*l MTT (5 mg/ml; Sigma) to each well and 5 h incubation at 37°C. Following this, the supernatant was aspirated and 450 *μ*l DMSO (Sigma)was added to each well. The ADSCs were then agitated for 15 min, prior to 200 *μ*l of the liquid being removed and transferred to a 96-well plate. An enzyme-linked immunosorbent assay detector was used to monitor the photometric value at a wavelength of 570 nm (A_570_). The A_570_ was proportional to the rate of cell proliferation. At each time-point, seven wells were used to determine the mean and standard deviation values for the two groups (experimental and control).

### Observation of cellular morphology through scanning electron microscopy (SEM)

Following the incorporation of the ADSCs into the SFCS for 2 and 6 days, the composite scaffold was removed. Subsequent to this, glutaraldehyde fixation, graded alcohol series dehydration, critical point drying and metal spraying were performed. SEM (TESCAN, Brno, Czech Republic) was then used to observe the adherence of the ADSCs onto the SFCS scaffold.

### Observation of cellular morphology through hematoxylin and eosin (H&E) staining

Following the incorporation of the ADSCs into the SFCS for 2, 8 and 10 days, the composite scaffold was removed and the cells were fixed in 4% polyformaldehyde (Xi’an chemical reagent factory). H&E staining was then conducted.

### Statistical analysis

Statistical analysis was performed using SPSS version 13.0 statistical software (SPSS, Inc., Chicago, IL, USA) and group comparisons were conducted using a t-test. All values are presented as the mean ± standard error (SE). P<0.05 was considered to indicate a statistically significant difference.

## Results

### Observation of ADSC morphology

**Figure 1.**
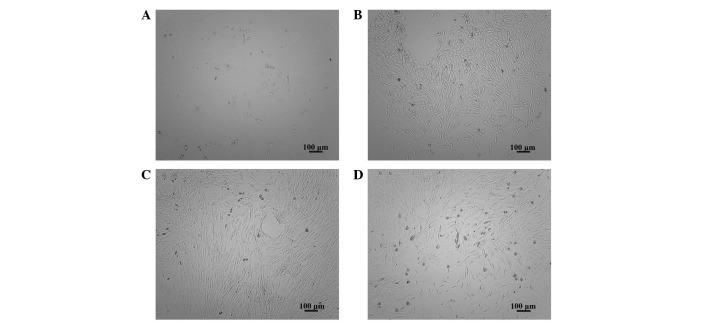
Morphology of the adipose tissue-derived stem cells (ADSCs). Morphology of: (A) ADSCs at the six-hour time point; (B) the primary culture at three days; (C) the primary culture at seven days; (D) the seventeenth generation. Magnification, ×100.

Six hours after inoculation of ADSCs, a few adherent cells were observed (Fig. 1A). At the 3-day time-point, an increase in the number of adherent cells was apparent (Fig. 1B), while at the 7-day time-point, the cells were observed to have reached 90% confluence (Fig. 1C). There were no marked differences in the morphology or proliferative characteristics between the seventeenth generation of cells and the primary cells (Fig. 1D).

### Induction of the ADSCs into osteogenic and adipogenic directions

**Figure 2.**
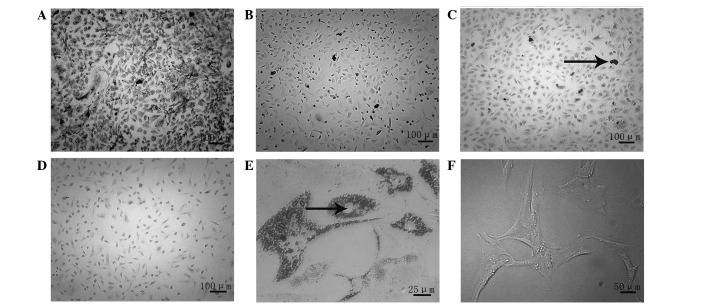
Osteogenic and adipogenic induction demonstrated by staining. Osteogenic induction at 2 weeks in the (A) experimental and (B) control groups [alkaline phosphatase (ALP) staining; magnification, ×100]. Osteogenic induction at 4 weeks, in the (C) experimental and (D) control groups (Alizarin Red staining; magnification, ×100). The arrow indicates calcified nodules. (E) Adipogenic induction at 2 weeks in the experimental group (Oil Red O staining; magnification, ×400). The arrow indicates fat droplets. (F) Adipogenic induction at 2 weeks in the control group (magnification, ×200).

Osteogenic induction was observed at 2 weeks, following alkaline phosphatase staining. In the experimental group, black particulate material was observed in the cytoplasm of the cells (Fig. 2A), while this was not evident in the control group (Fig. 2B). Alizarin Red staining at the 4-week time-point revealed the formation of calcium nodules in the experimental group (Fig. 2C), while this was not apparent in the control group (Fig. 2D). Oil Red O staining was performed at 2 weeks, in order to observe the adipogenic induction: The staining revealed the formation of fat droplets in the experimental group (Fig. 2E), whereas no fat droplets were observed in the control group (Fig. 2F).

### Observation of cellular morphology through SEM

**Figure 3.**
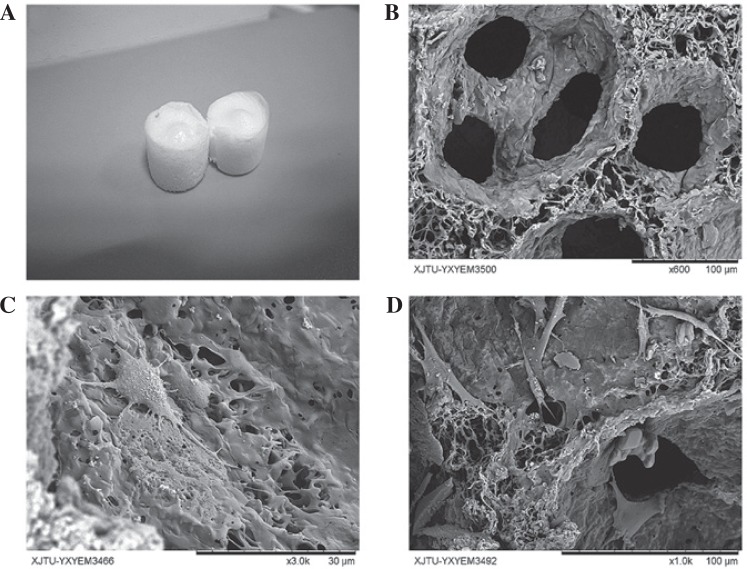
Observation of cell morphology through scanning electron microscopy (SEM). (A) Silk fibroin-chitosan scaffold (SFCS); (B) observation of the scaffold through SEM (magnification, ×600); (C) adipose tissue-derived stem cell (ADSC) morphology following 2 days of culture with the SFCS (magnification, ×3,000); (D) ADSC morphology following 6 days of culture with the SFCS (magnification, ×1,000).

The SFCS was made into a cylindrical shape (Fig. 3A) and observation of the SFCS using SEM revealed the pore diameter to be 80–120 *μ*m (Fig. 3B). At the 2-day time-point, it was noted that a few cells had adhered to the scaffold, although the cellular morphology was not entirely extended. A low level of matrix secretion was observed (Fig. 3C). At the 6-day time-point, the cell number appeared to have significantly increased compared with that at the 2-day time-point, and the cells displayed the appropriate, fully extended morphology with a growth-like spindle shape (Fig. 3D).

### Observation of cellular morphology using H&E staining

**Figure 4.**
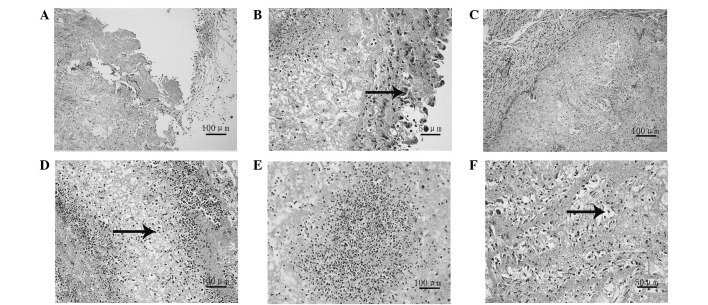
Observation of cellular morphology through hematoxylin and eosin (H&E) staining. (A and B) Cell morphology at two days (magnification, ×200 and ×400 respectively). The arrow indicates the cells on the silk fibroin-chitosan scaffold (SFCS) surface. (C) Morphology at six days (magnification, ×200); (D) morphology at eight days (magnification, ×200). The arrow indicates the cells moving into the interior of the scaffold. (E and F) Cell morphology at ten days [magnification, ×200 and ×400, respectively). The arrow indicates the cell distribution in the porous scaffold.

At the 2-day time-point, it was observed that a few cells had adhered on to the scaffold (Fig. 4A), with the majority of the cells located on the surface (Fig. 4B). At the 6-day time-point, the number of cells had increased compared with that at the 2-day time-point; however, the cells remained on the surface (Fig. 4C). At the 8-day time-point the cells were observed to have migrated from the surface of the scaffold into the interior (Fig. 4D), while at 10 days, it was noted that there was a large number of cells distributed uniformly in the inner scaffold (Fig. 4E and F).

### Cell adhesion rate

At the 6-hour time-point, 23.87% of the cells were observed to have adhered on the scaffold in the experimental group, compared with 32.19% adhered to the plate in the control group. This was a significant difference between the two groups; however, at the 12, 18 and 24-hour time-points, the cell adhesion rate in the experimental group was consistent with that of the control group and no significant difference was observed (Table I). These results suggested that the ADSCs and SFCS demonstrated good biocompatibility.

### Cellular proliferation rate

The results of the MTT assay revealed that the ADSCs were able to grow and proliferate effectively on the SFCS. At the 2 and 4-day time-points, a significant difference was observed between the control and experimental groups, and the cell proliferation of the control group was greater that of the experimental group; however, at the 6, 8 and 10-day time-points, no significant differences were identified between the two groups (Table II).

## Discussion

The basic method utilized when using tissue engineering to repair SCI is as follows (15): The cells are cultured at high concentration *in vitro*, amplified and adhered to the scaffold, prior to the scaffold being implanted into the spinal cord trauma cavity. Once in place, the implanted cells continue to proliferate in conjunction with the biological degradation and absorption of the scaffold. Thus, a new organization with the appropriate function and morphology is formed, ultimately resulting in the repair of the SCI and the reconstruction of its function. Consequently, the selection of a scaffold material and seed cells with good biocompatibility directly affects the outcome of the repair effort (16,17).

ADSCs display no specific surface antigen (18,19), and, therefore, the method of reductio ad absurdum was initially adopted, in order to determine the characteristics of the ADSCs. The assumption was that the separated cells were stem cells, and that, as such, had the potential for multilineage differentiation. Using third generation cells, the cells were induced into an osteogenic direction, and the cellular morphology was observed to change from fibroblast-like long spindles to square, polygonal or other forms. Two weeks subsequent to the induction, the alkaline phosphatase staining of the experimental group was positive, and large black particulate material appeared within the cell plasma; 4 weeks following the induction, the Alizarin Red staining of the experimental group was positive and there were evident square or irregular calcium salt deposits. This indicated that the cells had differentiated into osteoblasts and were exhibiting the corresponding characteristics. Two weeks subsequent to the adipogenic induction, Oil Red O staining revealed the formation of lipid droplets in the cytoplasm in the experimental group, whereas no lipid droplets were observed in the control group. This suggested that the ADSCs had differentiated into adipocytes. These results confirmed that the cells demonstrated the potential for multilineage differentiation and, in combination with the fact that the cells were extracted from the adipose tissue, determined that the cells were ADSCs.

SF is a natural structural protein that demonstrates no physiological activity and that has desirable biocompatibility and degradation characteristics. When implanted *in vivo*, SF elicits a mild inflammatory reaction; however, it is widely used in the field of tissue engineering (20,21). CS is the only alkaline polysaccharide in nature and it also exhibits a good biocompatibility (22). A previous demonstrated that it was possible to make a cylindrical and porous SFCS using a freezing drying method, and that it not only improved the brittle nature of SF in dry environments, but also reduced the cellular inhibitory effects displayed by CS (23).

The incorporation of the third generation cells into the scaffold and the assessment of the cell adhesion rate revealed that there was a significant difference between the experimental and control groups following 6 h of incorporation (P<0.05). However, as time progressed, i.e. following 12, 18 and 24 h, no significant difference was observed between the two groups, which indicated that there was a favorable adhesion characteristic between them. The examination of cellular proliferation on the scaffold revealed that there was a statistically significant difference between the experimental and control groups 2 and 4 days subsequent to the commencement of the incorporation process. This may have been due to a process of adaptation occurring following the initial incorporation of the ADSCs into the scaffold. The fact that the difference between the two groups at 4 days was less than the difference at 2 days may have been due to the ADSCs demonstrating an initial slow rate of proliferation on the scaffold, followed by a return to a more normal proliferation rate two days subsequently. At the 6, 8 and 10-day time-points, no significant differences were observed between the two groups (P>0.05). This indicated that there was good adhesion and biocompatibility between the ADSCs and the SFCS. Using SEM to examine the cellular morphology, it was observed that a few cells were composite with the scaffold following 2 days of incorporation and that the cellular morphology was not completely spindle-like. However, at the 6-day time-point, there was a significant increase in the cell number compared with that at the 2-day time-point and the cellular morphology was appropriate. H&E staining results revealed that, at the 2-day time-point, there were a few cells on the scaffold and that the majority of them were located on the surface of the scaffold, while at 6 days, the number of the cells was greater than at 2 days, although the cells remained on the surface. At the 8-day time-point, the cells had migrated from the surface into the interior of the scaffold and at 10 days, it was observed that there were a large number of cells distributed uniformly across the interior of the scaffold. These results were consistent with the results from the assessment of cellular proliferation

In this study, ADSCs were obtained and the multidifferentiative potential of the cells was demonstrated. Following this, experiments were used to reveal that there were desirable adhesion and biocompatibility properties between the ADSCs and the SFCS. The results indicated the potential of ADSCs as seed cells and SFCS as a scaffold material for use in the repair of SCI. This conclusion is likely to provide a foundation for further investigations.

## Figures and Tables

**Table I.** Cell adhesion rate of the experimental and control groups (n=7).

Time (h)	Experimental group (%)	Control group (%)	t value	P-value
6	25.07±0.63^a^	32.38±0.83^a^	−18.469	<0.05
12	51.46±1.21	52.49±1.13	−1.657	>0.05
18	76.21±1.33	77.34±1.30	−1.604	>0.05
24	93.98±1.07	94.47±1.68	−0.651	>0.05

Results are presented as the mean ± standard error.

a P<0.05, significant difference between control and experimental groups.

**Table II.** Photometric value (A_570_) of the experimental and control groups (n=7).

Time (days)	Experimental group	Control group	t value	P-value
2	0.3202±0.0024^a^	0.3473±0.0048^a^	−13.435	<0.05
4	0.4135±0.0092^a^	0.4224±0.0010^a^	−2.563	<0.05
6	0.7132±0.0003	0.7160±0.0062	−1.213	>0.05
8	0.7403±0.0070	0.7448±0.0045	−1.428	>0.05
10	0.7534±0.0068	0.7605±0.0073	−1.903	>0.05

Results are presented as the mean ± standard error.

a P<0.05, significant difference between control and experimental groups.

## References

[1] Madigan NN, McMahon S, O’Brien T, Yaszemski MJ, Windebank AJ (2009). Current tissue engineering and novel therapeutic approaches to axonal regeneration following spinal cord injury using polymer scaffolds. Respir Physiol Neurobiol.

[2] Cadotte DW, Fehlings MG (2011). Spinal cord injury: a systematic review of current treatment options. Clin Orthop Relat Res.

[3] Johnson PJ, Parker SR, Sakiyama-Elbert SE (2009). Controlled release of neurotrophin-3 from fibrin-based tissue engineering scaffolds enhances neural fiber sprouting following subacute spinal cord injury. Biotechnol Bioeng.

[4] Silva NA, Salgado AJ, Sousa RA (2010). Development and characterization of a novel hybrid tissue engineering-based scaffold for spinal cord injury repair. Tissue Eng Part A.

[5] Anghileri E, Marconi S, Pignatelli A (2008). Neuronal differentiation potential of human adipose-derived mesenchymal stem cells. Stem Cells Dev.

[6] Yang LY, Liu XM, Sun B, Hui GZ, Fei J, Guo LH (2004). Adipose tissue-derived stromal cells express neuronal phenotypes. Chin Med J (Engl).

[7] Luangbudnark W, Viyoch J, Laupattarakasem W, Surakunprapha P, Laupattarakasem P (2012). Properties and biocompatibility of chitosan and silk fibroin blend films for application in skin tissue engineering. Scientific World Journal.

[8] Cao Y, Wang B (2009). Biodegradation of silk biomaterials. Int J Mol Sci.

[9] Liu TL, Miao JC, Sheng WH (2010). Cytocompatibility of regenerated silk fibroin film: a medical biomaterial applicable to wound healing. J Zhejiang Univ Sci B.

[10] Jayakumar R, Prabaharan M, Sudheesh Kumar PT, Nair SV, Tamura H (2011). Biomaterials based on chitin and chitosan in wound dressing applications. Biotechnol Adv.

[11] Kim IY, Seo SJ, Moon HS (2008). Chitosan and its derivatives for tissue engineering applications. Biotechnol Adv.

[12] Zhang X, Yang D, Nie J (2008). Chitosan/polyethylene glycol diacrylate films as potential wound dressing material. Int J Biol Macromol.

[13] Chen L, Zhu Y, Li Y, Liu Y, Yu J (2011). Progress and prospect of electrospun silk fibroin in construction of tissue-engineering scaffold. Sheng Wu Gong Cheng Xue Bao.

[14] She Z, Jin C, Huang Z, Zhang B, Feng Q, Xu Y (2008). Silk fibroin/chitosan scaffold: preparation, characterization, and culture with HepG2 cell. J Mater Sci Mater Med.

[15] Johnson PJ, Parker SR, Sakiyama-Elbert SE (2010). Fibrin-based tissue engineering scaffolds enhance neural fiber sprouting and delay the accumulation of reactive astrocytes at the lesion in a subacute model of spinal cord injury. J Biomed Mater Res A.

[16] Dittmar R, Potier E, van Zandvoort M, Ito K (2012). Assessment of cell viability in three-dimensional scaffolds using cellular auto-fluorescence. Tissue Eng Part C Methods.

[17] Valarmathi MT, Yost MJ, Goodwin RL, Potts JD (2008). The influence of proepicardial cells on the osteogenic potential of marrow stromal cells in a three-dimensional tubular scaffold. Biomaterials.

[18] Choi YS, Dusting GJ, Stubbs S (2010). Differentiation of human adipose-derived stem cells into beating cardiomyocytes. J Cell Mol Med.

[19] Zuk PA (2010). The adipose-derived stem cell: looking back and looking ahead. Mol Biol Cell.

[20] Kasoju N, Bhonde RR, Bora U (2009). Preparation and characterization of *Antheraea assama* silk fibroin based novel non-woven scaffold for tissue engineering applications. J Tissue Eng Regen Med.

[21] Yin GB, Zhang YZ, Wang SD, Shi DB, Dong ZH, Fu WG (2010). Study of the electrospun PLA/silk fibroin-gelatin composite nanofibrous scaffold for tissue engineering. J Biomed Mater Res A.

[22] Dhandayuthapani B, Krishnan UM, Sethuraman S (2010). Fabrication and characterization of chitosan-gelatin blend nano-fibers for skin tissue engineering. J Biomed Mater Res B Appl Biomater.

[23] Gupta V, Davis G, Gordon A (2010). Endothelial and stem cell interactions on dielectrophoretically aligned fibrous silk fibroin-chitosan scaffolds. J Biomed Mater Res A.

